# Victimization Experiences and Mental Health Outcomes Among Grades 7 to 12 Students in Manitoba, Canada

**DOI:** 10.1007/s42380-019-00056-0

**Published:** 2019-12-02

**Authors:** Ashley Stewart-Tufescu, Samantha Salmon, Tamara Taillieu, Janique Fortier, Tracie O. Afifi

**Affiliations:** 1 Departments of Community Health Sciences and Psychiartry, Max Rady College of Medicine, Rady Faculty of Health Sciences, University of Manitoba, PZ-449, PsychHealth Centre, Health Sciences Centre 771 Bannatyne Avenue, Winnipeg, Manitoba R3E 3N4 Canada; 2 Department of Community Health Sciences, University of Manitoba, Winnipeg, Canada; 3 Departments of Community Health Sciences and Psychiartry, Max Rady College of Medicine, Rady Faculty of Health Sciences, University of Manitoba, S113-750 Bannatyne Avenue, Winnipeg, Manitoba R3E 0W5 Canada

**Keywords:** Victimization, Bullying, Cyberbullying, Mental health functioning, Sadness/hopelessness, Adolescents

## Abstract

Victimization experiences, including traditional forms of bullying, discriminatory harassment, and cyber victimization, are associated with numerous detrimental consequences in adolescence and over the life course. The objective of the current study was to understand the relationships between nine experiences of victimization and mental health outcomes among students in grades 7 to 12 in Manitoba, Canada. Data were drawn from the 2012–2013 Manitoba Youth Health Survey (*N* = 64,174; response rate = 67%). Mental health outcomes included mental health functioning and emotional well-being, and feelings of sadness and hopelessness. The prevalence of moderate/languishing mental health functioning and emotional well-being ranged from 35.2% (boys in grades 7 to 9) to 51.0% (girls in grades 10 to 12). The prevalence of feeling sad and hopeless ranged from 31.4% (boys in grades 7 to 9) to 57.7% (girls in grades 10 to 12). All nine victimization types were associated with increased odds of having moderate/languishing mental health functioning and emotional well-being and feeling sad and hopeless for both boys and girls in grades 7 to 9 and 10 to 12, although some gender and grade differences were noted. A dose-response trend was found with increased odds of moderate/languishing mental health functioning and emotional well-being corresponding with increased frequency of being victimized. A similar trend was noted for girls only for feeling sad and hopeless. Effective prevention and intervention strategies targeting boys and girls and across grades 7 to 12 are needed to improve mental health functioning and emotional well-being, and reduce feelings of sadness and hopelessness among adolescents with victimization experiences.

## Introduction

Victimization experiences, including bullying, discriminatory harassment, and cyber victimization are relatively common among adolescents in North America, and are associated with numerous detrimental outcomes in adolescence and adulthood. While prevalence estimates of victimization experiences vary, in Canada, previous research has reported that approximately 63% of youth experienced bullying at least once over a 2-month period, and notably, this estimate remained relatively stable over a 2-year period (Freeman et al. 2011). Other multinational research has reported overall prevalence estimates of adolescent bullying victimization upwards of 40% (Elgar et al. 2015; Modecki et al. 2014). Differences in prevalence estimates may vary due to how victimization experiences are operationalized and measured, as well as the study population of interest and sample characteristics (Söderberg and Björkqvist 2019). Bullying is traditionally defined according to three main characteristics: hostile intent, repetition, and power differential. A commonly used definition of bullying is a repeated aggressive behaviour towards another person occurring in a variety of forms, with hostile intent from the perpetrator to the victim, and includes an observed or perceived power imbalance (Freeman et al. 2011; Gladden et al. 2014; Olweus 1996). Furthermore, bullying is not isolated to the school environment; bullying victimization can occur in many settings and present in many forms, such as physical force, comments in either written or verbal form, and relational behaviours to harm reputation or relationships (Gladden et al. 2014).

In recent years, given the expansion in peer victimization research, the reliance on the operationalization of a more traditional definition of bullying victimization has become an area of debate (Turner et al. 2015). Specifically, there is increasing recognition that bullying experiences fit within the context of broader experiences of victimization, which also encompass experiences of cyber victimization, discriminatory harassment (e.g. discrimination based on sexual orientation, body shape, or race), and other forms of peer aggression (Salmon et al. 2018; Söderberg and Björkqvist 2019). Along with a growing recognition of these broader experiences of victimization beyond the traditional forms of bullying, others are making the argument that experiences of victimization do not necessarily need to be *repetitive* for them to be detrimental to the victim, nor do the experiences need to occur in the context of a power imbalance between a perpetrator and a victim (Finkelhor et al. 2012). For example, given the nature of cyber victimization, it may be the case that a single incident of online peer aggression triggers a ripple effect that further compounds the aggression for the victim with long-lasting detrimental consequences (Patchin and Hinduja 2015; Volk et al. 2014; Salmon et al. 2018). Taken together, there is a need to move beyond a reliance on general global measures of bullying based on traditional definitions, and expand our operationalization of contemporary and multiple experiences of victimization that includes traditional forms of bullying, discriminatory harassment, and more contemporary experiences of cyber victimization.

## Victimization Experiences and Mental Health

Previous research, including a recent systematic review and meta-analysis, has established a robust association between global assessments of bullying and a number of detrimental mental health outcomes in adolescence and over the life course (Arseneault 2017; Haraldstad et al. 2016; Moore et al. 2017; Troop-Gordon 2017). For example, bullying victimization is associated with self-harming behaviour (Bucchianeri et al. 2014), lower self-esteem and quality of life (Bucchianeri et al. 2014; Haraldstad et al. 2016), depressive symptoms (Bucchianeri et al. 2014; Cole et al. 2016; Fahy et al. 2016; Gámez-Guadix et al. 2013; Hill et al. 2017; Lemstra et al. 2012; Puhl and Luedicke 2012; Turner et al. 2013), anxiety symptoms (Fahy et al. 2016), substance use (Mackie et al., 2011; Radliff et al. 2012; Turner et al. 2018), suicidal behaviours (Litwiller and Brausch 2013; Moore et al. 2017; Skapinakis et al. 2011), and mental health service use (Evans-Lacko et al. 2017). As well, previous research has indicated that experiences of bullying in childhood and adolescence are related to an increased likelihood of mental disorders in adulthood (Copeland et al. 2013; Lund et al. 2008; Sigurdson et al. 2014; Takizawa et al. 2014).

What remains unknown is how different specific experiences of victimization are related to negative mental health outcomes in adolescence and adulthood. As mentioned, bullying research often includes general or global assessments of any bullying or is limited to more traditional forms of face-to-face bullying experiences such as being physically taunted or ridiculed based on intentionality, repetition, and power imbalances (Gladden et al. 2014; Olweus 1993; Solberg et al. 2007). More recent research has begun to examine less traditional forms of victimization including cyberbullying (Fahy et al. 2016; Fisher et al. 2016; Hill et al. 2017; Litwiller and Brausch 2013; Merrill and Hanson 2016) and forms of discriminatory harassment based on body composition, sexual identity and orientation, and race and ethnicity (Bucchianeri et al. 2014; Carey et al. 2018; Goebert et al. 2011; Johns et al. 2017; Juvonen and Graham 2014; Kahle and Peguero 2017; Mustanski et al. 2016; Puhl et al. 2016). However, there remains a dearth of research that has examined multiple individual types of victimization related to poor mental health outcomes in adolescence. As well, it is currently unknown how the relationships between specific types of victimization experiences and mental health outcomes may vary among boys and girls and also by adolescents in younger and older grade levels. Previous research indicates that bullying victimization peaks between the ages of 12 and 15 years and then decreases by the age of 18 years (Hymel and Swearer 2015). Similar results were found in a recent study of the prevalence of bullying among Korean youth between the ages of 10 and 17 in grades 6 to 10, suggesting that bullying is less common in higher school grades and decreases with age. In 6th grade (ages 10 to 12 years), nearly 10% of Korean students experienced bullying victimization compared to just over 6% of students in 10th grade (ages 15 to 17 years) (Seo et al. 2017).

Previous research has also noted gender differences in experiences of bullying victimization, other victimization experiences, and other outcomes (Cook et al. 2010; Turner et al. 2018). While not specific to peer victimization, sex differences in externalizing and internalizing behaviour symptoms among youth that have witnessed victimization and maltreatment have been noted (Maschi et al. 2008). Similarly, an examination of exposure to bullying, reactions, and psychological adjustment among children between the ages of 11 and 15 years found that adolescent girls scored higher on internalizing behaviours symptoms, whereas boys scored higher on externalizing behaviours symptoms (Nabuzoka et al. 2009).

A more recent study of sex differences, childhood bullying, and substance use found that victimized males and females differed in their use of substances in adolescence and adulthood compared to those not victimized (Connolly 2017). In this study, victimized males were more likely to engage in quicker uptake of nicotine and marijuana and slower declines of their use, whereas victimized females were more likely to engage in quicker uptake of nicotine and slower declines of alcohol and cigarette use from adolescence into adulthood compared to non-victimized youth. Similarly, Turner et al. (2018) reported a difference in bullying victimization and illicit drug use types by sex, finding a stronger association between any bullying victimization and illicit drug use among girls compared to boys. In another study, Williams et al. (2017) compared experiences of cyberbullying and physical bullying along with depressive symptoms and suicidal behaviours among adolescents transitioning to high school (entering 9th grade). The authors found that girls were significantly more likely to be victims of verbal and social victimization and cyberbullying compared to boys, whereas there were no significant sex differences in experiences of physical bullying victimization. Ninth grade boys that reported being physically victimized were more likely to experience depressive symptoms than non-victimized boys, and both boys and girls with experiences of verbal and social victimization were more likely to experience depressive symptoms than non-victimized peers. Moreover, ninth grade girls with a history of cyber victimization were more likely to report depressive symptoms and suicidal behaviours. What remains unknown is if the relationships between specific types of victimization experiences and poor mental health outcomes among adolescents follow this trend for gender- and grade-level differences.

Ongoing research is needed to better understand how specific types of victimization experiences are related to poor mental health outcomes, specifically among adolescent boys and girls in lower and higher school grades. More detailed information related to experiences of multiple victimization types and mental health outcomes may provide evidence to inform, refine, and tailor victimization prevention and intervention strategies. Therefore, a more in-depth understanding of multiple specific experiences of victimization, including traditional forms of bullying, forms of discriminatory harassment, and contemporary cyber victimization experiences during adolescence, is warranted.

The purpose of the current study was to address the above-mentioned limitations in the literature in a number of ways. First, this study examined nine experiences of victimization rather than an examination of a general global assessment of any bullying/victimization. Furthermore, there has been a call for more research on discrimination- or harassment-based victimization (Juvonen and Graham 2014), which are included in the current study as victimization experiences specific to race or culture, gender identity or sexual orientation, and body composition, size, or appearance, and more contemporary experiences of cyber victimization that are thought to operate differently than the face-to-face bullying victimization (Byers et al. 2019). Second, the sample size is large (*N* = 64,174), which provides a unique opportunity to examine the data with a level of detail that is not usually possible due to statistical power issues. This allows for the investigation of nine victimization items using an ordinal scale of increasing frequency rather than general dichotomized ‘yes’ or ‘no’ categories. This more sensitive approach provides the opportunity for the examination of dose-response trends based on the frequency of each of the nine victimization experiences. Additionally, the large sample size enables all analyses to be stratified by both gender and grade categories so that relationships between the nine victimization experiences and mental health outcomes can be understood separately among boys and girls in younger (grades 7 to 9) and older (grades 10 to 12) grades.

Therefore, the main objectives of the current study were to determine if (1) experiences of nine victimization types increase the likelihood of poor mental health outcomes including (a) moderate/languishing mental health functioning and emotional well-being and (b) feelings of sadness and hopelessness among boys and girls in grades 7 to 9 (on average 11 to 14 years of age) and grades 10 to 12 (on average 15 to 18 years of age) in Manitoba; and to determine if (2) a dose-response trend was noted with increasing frequency of nine victimization experiences corresponding with increased odds of (a) moderate/languishing mental health functioning and emotional well-being and (b) feelings of sadness and helplessness. Based on the literature reviewed, we hypothesized that all nine victimization experiences would be associated with poorer mental health outcomes and that a dose-response effect would exist and indicate that increased frequencies of victimization experiences would relate to increased odds of negative mental health outcomes. We also hypothesized that there would be notable gender and grade-level differences in the association between the nine victimization types and the mental health outcomes; specifically, girls and students in younger grades were expected to have larger effects.

## Method

### Data and Sample

Data were drawn from the cross-sectional 2012–2013 Youth Health Survey (YHS second cycle). The YHS is a self-report, pencil-and-paper classroom-based survey offered in either French or English. The YHS includes 12 sections related to students’ health, including questions about physical activity, eating, sleep, safety, work and volunteering, sexuality, substance use, and bullying victimization. Additional information about the content of the YHS is available elsewhere (Partners in Planning for Health Living 2013). The YHS was administered to adolescents in grades 7 to 12 (ages 11 to 18 years) at public, independent, Colony, Francophone, and First Nations schools throughout the province of Manitoba in Canada. All schools with students in the eligible grades were invited to join the study; 73% of eligible schools participated in the YHS survey. Participants in the younger grades (i.e. grades 7 to 9) were between 11 and 14 years of age, whereas students in the older grades (i.e. grades 10 to 12) were between 15 and 18 years of age. The response rate for the YHS was 67%, with 64,174 students of the total 95,659 students registered in the 2012–2013 academic year in Manitoba schools completing the YHS. School recruitment and administration of the YHS was supported by Partners in Planning for Healthy Living, a network of government and nongovernment organizations throughout Manitoba and all Manitoba Regional Health Authorities (Partners in Planning for Healthy Living 2013). Each school division was responsible for notifying parents/guardians about the study. Depending on the school division, active parental consent (i.e. the parent/guardian is required to sign a consent form and send it back to the school) or passive parental consent (i.e. the parent/guardian is provided with an opportunity to opt their child out of being invited to provide their assent to participate; if the parent/guardian does not opt-out then consent is implied) was required. Once parental consent was established, student assent was required prior to the student completing the YHS. Student participation was voluntary, and if the student chose not to participate, they remained in the classroom and worked independently throughout the duration of the survey. The Health Research Ethics Board at the University of Manitoba granted ethics approvals to conduct the present study.

### Measurement

#### Victimization Experiences

Nine types of victimization experiences were assessed with the YHS. Recently published research has conceptualized and examined experiences of victimization using these nine YHS items and has demonstrated reliable psychometric properties (Turner et al. 2018; Salmon et al. 2018). In this study, respondents reported how often in the past 12 months they experienced the following types of victimization: (1) were physically threatened or injured; (2) were threatened or injured with a weapon such as a gun, knife, or club; (3) were bullied, taunted, or ridiculed; (4) someone said something bad about their race or culture; (5) someone said something bad about their sexual orientation or gender identity; (6) someone said something bad about their body shape, size, or appearance; (7) someone asked for personal information over the internet (e.g. address, phone number, or last name); (8) someone made them feel unsafe when in contact with them over the internet; and (9) someone bullied or picked on them through the internet (e.g. posted something on Facebook or emailed them). The Cronbach’s alpha of these nine items is 0.77, demonstrating adequate to good internal consistency. The frequency of each victimization experience was reported by the respondent using the following categories: 'never', '1 to 5 times', '6 or more times', and 'every day'. Assessing the frequency of each victimization experience also provides an indication of the severity of victimization events.

#### Mental Health and Well-Being

Mental health functioning and emotional well-being in the past month (30 days) was measured using the 14-item Mental Health Continuum-Short Form (MHC-SF) based on the work of Corey Keyes that assesses psychological, social, and emotional functioning and well-being (Keyes 2002; Keyes et al. 2008; Lamers et al. 2011; Westerhof and Keyes 2010). The MHC-SF is derived from the long-form version of the Mental Health Continuum (MHC-LF). Both the MHC-SF and the MHC-LF have demonstrated high estimates of internal consistency reliability (alphas greater than 0.80) and have been validated and used in hundreds of studies of mental health and well-being (Keyes 2009a) with diverse nationally representative samples including adolescents between the ages of 12 and 18 years (Keyes 2005, 2009b).

The MHC-SF includes constructs such as happiness; interest in or satisfaction with life; contributions to and feelings about society, belonging to a community; personal abilities; and relationships. Algorithms developed for this tool were applied to place respondents as having flourishing mental health (i.e. high positive mental health functioning and high emotional well-being), moderate mental health (i.e. high positive mental health functioning and low emotional well-being or low positive mental health functioning and high emotional well-being), and languishing mental health (low positive mental health functioning and low emotional well-being). The moderate and languishing categories were combined into a dichotomous variable for the current analysis and then used compared to flourishing mental health and emotional well-being (i.e. flourishing vs. moderate/languishing). This dichotomized approach was taken for two reasons. First, we were interested in comparing optimal mental health (i.e. flourishing mental health) to anything less than optimal (i.e. moderate and languishing mental health). Second, we wanted to ensure the reliability of the model estimates once stratified by gender, grade, and frequency of types of victimization, including in daily experiences.

#### Sadness and Hopelessness

Self-reported feelings of sadness and hopelessness were assessed by a single item from Section 3 of the YHS (Partners in Planning for Healthy Living 2013). Respondents were asked, ‘during the past 12 months, did you ever feel so sad or hopeless that you stopped doing some usual activities for a while?’. Response categories included ‘yes’ or ‘no’. This item has also been used to assess sadness and hopelessness in recently published research (Turner et al. 2018).

## Statistical Analysis

For all analyses, data were stratified by gender (i.e. boys or girls) and grade level (i.e. grades 7 to 9 and grades 10 to 12). First, the prevalence of mental health functioning and emotional well-being and feeling sad and hopeless was computed using crosstabulations with chi-squared (*X*^2^) tests of association. Second, logistic regression models were computed to determine if each victimization type was associated with increased odds of poorer (moderate/languishing) mental health functioning and emotional well-being separately among boys and girls in grades 7 to 9 and grades 10 to 12. Finally, logistic regression analyses were computed to determine if each victimization type was associated with increased odds of feeling sad and hopeless separately among boys and girls in grades 7 to 9 and grades 10 to 12. In all logistic regression models, the four-category victimization variable (‘never’, ‘1–5 times’, ‘6 or more times’, ‘every day’) was entered as the independent variable, and the mental health outcome variable was entered as the dependent variable. The ‘never’ category of victimization variable is the reference category in all the models.

## Results

The prevalence of mental health functioning and emotional well-being and feelings of sadness or hopelessness among boys and girls in grades 7 to 9 and grades 10 to 12 is presented in Table 1. The prevalence of moderate/languishing mental health functioning and emotional well-being ranged from 35.2% (boys in grades 7 to 9) to 51.0% (girls in grades 10 to 12). Among boys and girls, the prevalence of moderate/languishing mental health functioning and emotional well-being was higher in grades 10 to 12 compared to grades 7 to 9. Similarly, the prevalence of feeling sad and hopeless ranged from 31.4% (boys in grades 7 to 9) to 57.7% (girls in grades 10 to 12) with the highest prevalence being reported in higher grades.

**Table 1:** Prevalence of mental health outcomes among boys and girls in grades 7 to 9 and 10 to 12 in Manitoba

Mental health outcomes	Boys		Girls		*X*^2^ (df)
	Grade 7–9 (n = 17,872)	Grade 10–12 (n = 15,040)	Grade 7–9 (n = 16,768)	Grade 10–12 (n = 14,494)	
	% (n)	% (n)	% (n)	% (n)	
Mental health functioning and emotional well-being					
Flourishing	64.8 (11,344)	56.9 (8420)	58.5 (9689)	49.0 (7032)	811.9 (3)***
Moderate/languishing	35.2 (6161)	43.1 (6378)	41.5 (6874)	51.0 (7310)	
Feel sad and hopeless					
No	68.6 (11,814)	60.2 (8825)	53.7 (8755)	42.3 (6007)	2330.3 (3)***
Yes	31.4 (5402)	39.8 (5831)	46.3 (7553)	57.7 (8193)	

***p < 0.001

The associations between each victimization type and moderate/languishing mental health functioning and emotional well-being among boys and girls in grades 7 to 9 and grades 10 to 12 are reported in Table 2. In the models, all victimization types were associated with increased odds of having moderate/languishing mental health functioning and emotional well-being among boys and girls across grades 7 to 12. The largest effects were noted among girls in younger grades who experienced being threatened or injured with a weapon every day (odds ratio [OR] = 8.34, 95% confidence interval [CI] = 1.85–37.64); were bullied, taunted, or ridiculed every day (OR = 7.55, 95% CI = 6.13–9.31); having something bad said about their sexual orientation or gender identity every day (OR = 7.71, 95% CI = 4.81–12.37); having something bad said about their body shape, size, or appearance every day (OR = 9.91, 95% CI = 8.14–12.07); and being bullied over the internet every day (OR = 8.00, 95% CI = 5.76–11.12). With few exceptions, a dose-response trend was noted with increased frequency of each victimization type corresponding with higher odds of having moderate/languishing mental health functioning and emotional well-being for both boys and girls in all grade categories. For example, dose-response trends were noted for boys in grades 7 to 9 who experienced being physically threatened or injured (OR range = 1.67–4.21); for boys in grades 10 to 12 who were bullied, taunted, or ridiculed (OR range = 1.65–3.03); for girls in grades 7 to 9 who experienced being bullied, taunted, or ridiculed (OR range = 2.10–7.55); and for girls in grades 10 to 12 who were bullied or picked on through the internet (OR range = 1.93–6.79).

**Table 2:** The relationship between each victimization experience and moderate/languishing mental health functioning and emotional well-being among boys and girls in grade 7 to 12 in Manitoba

Type of victimization	Boys				Girls			
	Grade 7–9		Grade 10–12		Grade 7–9		Grade 10–12	
	OR (95% CI)	*p*-value	OR (95% CI)	*p*-value	OR (95% CI)	*p*-value	OR (95% CI)	*p*-value
Physically threatened or injured you								
Never	1.00	<0.001	1.00	<0.001	1.00	<0.001	1.00	<0.001
1–5 times	1.67 (1.55–1.81)		1.68 (1.55–1.82)		2.87 (2.64–3.13)		2.10 (1.93–2.29)	
6+ times	2.70 (2.31–3.16)		2.09 (1.76–2.48)		5.06 (4.09–6.26)		3.73 (2.95–4.72)	
Every day	4.21 (3.05–5.82)		2.46 (1.91–3.16)		4.76 (2.91–7.79)		4.51 (2.45–8.31)	
Threatened or injured you with a weapon such as a gun, knife or club								
Never	1.00	<0.001	1.00	<0.001	1.00	<0.001	1.00	<0.001
1–5 times	2.42 (2.10–2.78)		1.77 (1.56–2.00)		3.98 (3.27–4.85)		2.38 (1.95–2.91)	
6+ times	2.48 (1.71–3.59)		3.00 (2.15–4.18)		4.64 (2.68–8.03)		3.48 (1.88–6.47)	
Every day	4.70 (2.87–7.68)		2.41 (1.77–3.30)		8.34 (1.85–37.64)		4.53 (1.53–13.39)	
Bullied, taunted or ridiculed you								
Never	1.00	<0.001	1.00	<0.001	1.00	<0.001	1.00	<0.001
1–5 times	1.51 (1.40–1.64)		1.65 (1.53–1.79)		2.10 (1.95–2.26)		1.81 (1.68–1.96)	
6+ times	2.49 (2.21–2.80)		2.20 (1.93–2.50)		3.93 (3.52–4.39)		3.43 (3.03–3.89)	
Every day	4.12 (3.32–5.10)		3.03 (2.47–3.72)		7.55 (6.13–9.31)		6.86 (5.08–9.27)	
Said something bad about your race or culture								
Never	1.00	<0.001	1.00	<0.001	1.00	<0.001	1.00	<0.001
1–5 times	1.46 (1.33–1.60)		1.44 (1.31–1.58)		1.89 (1.72–2.06)		1.57 (1.43–1.72)	
6+ times	2.09 (1.78–2.46)		1.85 (1.59–2.16)		3.21 (2.70–3.82)		2.10 (1.76–2.50)	
Every day	2.94 (2.28–3.79)		2.09 (1.72–2.54)		4.73 (3.37–6.64)		3.96 (2.72–5.77)	
Said something bad about your sexual orientation or gender identity								
Never	1.00	<0.001	1.00	<0.001	1.00	<0.001	1.00	<0.001
1–5 times	1.96 (1.72–2.22)		1.82 (1.60–2.07)		2.46 (2.18–2.77)		2.10 (1.85–2.38)	
6+ times	3.16 (2.51–3.97)		2.18 (1.74–2.74)		4.96 (3.88–6.35)		2.82 (2.24–3.56)	
Every day	3.93 (2.82–5.49)		2.33 (1.82–3.00)		7.71 (4.81–12.37)		5.83 (3.42–9.95)	
Said something bad about your body shape, size or appearance								
Never	1.00	<0.001	1.00	<0.001	1.00	<0.001	1.00	<0.001
1–5 times	1.55 (1.43–1.69)		1.67 (1.54–1.82)		2.22 (2.06–2.39)		1.87 (1.74–2.01)	
6+ times	2.67 (2.33–3.05)		2.12 (1.84–2.44)		4.46 (4.00–4.99)		3.13 (2.79–3.51)	
Every day	4.17 (3.35–5.18)		2.93 (2.39–3.60)		9.91 (8.14–12.07)		6.03 (4.78–7.61)	
Asked for personal information over the internet (e.g. address, phone # or last name)								
Never	1.00	<0.001	1.00	<0.001	1.00	<0.001	1.00	<0.001
1–5 times	1.31 (1.18–1.46)		1.44 (1.31–1.59)		2.10 (1.93–2.29)		1.55 (1.43–1.69)	
6+ times	1.99 (1.60–2.46)		1.63 (1.36–1.96)		2.99 (2.52–3.54)		2.16 (1.86–2.51)	
Every day	3.07 (2.06–4.57)		2.24 (1.71–2.94)		4.34 (2.93–6.43)		3.90 (2.51–6.04)	
Made you feel unsafe when you were in contact with them over the internet								
Never	1.00	<0.001	1.00	<0.001	1.00	<0.001	1.00	<0.001
1–5 times	1.94 (1.66–2.26)		1.90 (1.61–2.25)		2.48 (2.23–2.76)		1.93 (1.73–2.15)	
6+ times	3.29 (2.21–4.89)		2.32 (1.55–3.45)		4.03 (3.17–5.11)		2.31 (1.81–2.95)	
Every day	4.54 (2.80–7.37)		2.43 (1.75–3.37)		5.24 (3.24–8.47)		3.60 (2.06–6.30)	
Bullied or picked on you through the internet (e.g. posted something on Facebook or emailed you)								
Never	1.00	<0.001	1.00	<0.001	1.00	<0.001	1.00	<0.001
1–5 times	1.69 (1.49–1.92)		1.96 (1.71–2.23)		2.29 (2.09–2.51)		1.93 (1.75–2.13)	
6+ times	2.08 (1.60–2.71)		2.19 (1.66–2.89)		3.74 (3.21–4.37)		2.98 (2.48–3.59)	
Every day	5.75 (3.78–8.74)		2.44 (1.81–3.29)		8.00 (5.76–11.12)		6.79 (4.23–10.92)	

*Note.* OR = odds ratio; CI = confidence interval

Table 3 presents the associations between each victimization type and feelings of sadness or hopelessness among boys and girls in grades 7 to 9 and grades 10 to 12. All victimization types were associated with increased odds of reporting feelings of sadness or hopelessness to the point of stopping some usual activities. The largest effects were generally noted among girls in grades 7 to 9 who were physically threatened or injured six or more times (OR = 8.30, 95% CI = 6.42–10.73); were threatened or injured with a weapon six or more times (OR = 9.32, 95% CI = 4.46–19.49); were bullied, taunted, or ridiculed every day (OR = 11.76, 95% CI = 9.19–15.03); had something bad said about your sexual orientation or gender identity every day (OR = 16.79, 95% CI = 8.50–33.15) and your body shape, size, or appearance every day (OR = 14.54, 95% CI = 11.54–18.31); and were bullied or picked on via the internet every day (OR = 19.55, 95% CI = 12.07–31.60). The largest dose-response trends were noted primarily for girls in grades 7 to 9 that had experienced bullying, taunting, or ridiculing (OR range = 2.93–11.76); had something said about their race or culture (OR range = 2.64–7.11), sexual orientation or gender identity (OR range = 4.08–16.79), body shape, size, or appearance (OR range = 2.90–14.54); and had been bullied or picked on via the internet (OR range = 3.72–19.55). Two other notable dose-response trends were found among boys in grades 7 to 9 who were bullied, taunted, or ridiculed (OR range = 2.38–8.05) and girls in grades 10 to 12 who experienced bullying, truanting, and ridiculing (OR range = 2.52–9.72) and experienced someone saying something bad about their body shape, size, or appearance (OR range = 2.44–10.91). Dose-response trends were less clear for boys in grades 10 to 12; however, all victimization types, regardless of frequencies of occurrence, were associated with increased odds of feelings of sadness and hopelessness for boys across the grades.

**Table 3:** The relationship between each victimization type and feelings of sadness and hopelessness among boys and girls in grade 7 to 12 in Manitoba

Type of Victimization	Boys				Girls			
	Grade 7–9		Grade 10–12		Grade 7–9		Grade 10–12	
	OR (95% CI)	*p*-value	OR (95% CI)	*p*-value	OR (95% CI)	*p*-value	OR (95% CI)	*p*-value
Physically threatened or injured you								
Never	1.00	<0.001	1.00	<0.001	1.00	<0.001	1.00	<0.001
1–5 times	2.61 (2.41–2.83)		2.41 (2.23–2.61)		4.73 (4.31–5.20)		3.61 (3.27–3.98)	
6+ times	4.37 (3.72–5.13)		3.66 (3.06–4.38)		8.30 (6.42–10.73)		9.29 (6.61–13.06)	
Every day	4.61 (3.36–6.32)		2.54 (1.98–3.26)		6.34 (3.66–10.99)		5.05 (2.56–9.94)	
Threatened or injured you with a weapon such as a gun, knife, or club								
Never	1.00	<0.001	1.00	<0.001	1.00	<0.001	1.00	<0.001
1–5 times	3.59 (3.12–4.15)		2.63 (2.32–2.98)		6.98 (5.45–8.92)		5.73 (4.33–7.59)	
6+ times	4.53 (3.06–6.70)		3.64 (2.60–5.11)		9.32 (4.46–19.49)		8.33 (3.33–20.84)	
Every day	3.27 (2.08–5.14)		2.07 (1.52–2.81)		6.85 (1.52–30.89)		Not reported	
Bullied, taunted, or ridiculed you								
Never	1.00	<0.001	1.00	<0.001	1.00	<0.001	1.00	<0.001
1–5 times	2.38 (2.20–2.58)		2.26 (2.09–2.46)		2.93 (2.72–3.16)		2.52 (2.33–2.73)	
6+ times	4.15 (3.68–4.68)		3.49 (3.06–3.99)		5.94 (5.27–6.70)		5.43 (4.69–6.29)	
Every day	8.05 (6.14–10.11)		3.50 (2.85–4.29)		11.76 (9.19–15.03)		9.72 (6.76–13.98)	
Said something bad about your race or culture								
Never	1.00	<0.001	1.00	<0.001	1.00	<0.001	1.00	<0.001
1–5 times	2.36 (2.15–2.59)		2.27 (2.07–2.49)		2.64 (2.41–2.90)		2.30 (2.08–2.54)	
6+ times	3.39 (2.88–3.99)		2.63 (2.25–3.06)		5.50 (4.49–6.72)		4.26 (3.43–5.29)	
Every day	3.91 (3.03–5.05)		2.32 (1.91–2.82)		7.11 (4.76–10.62)		3.81 (2.56–5.68)	
Said something bad about your sexual orientation or gender identity								
Never	1.00	*<0*.001	1.00	<0.001	1.00	<0.001	1.00	<0.001
1–5 times	3.02 (2.66–3.44)		2.49 (2.18–2.83)		4.08 (3.57–4.67)		2.89 (2.51–3.33)	
6+ times	4.14 (3.27–5.25)		3.63 (2.85–4.61)		8.08 (5.97–10.92)		5.89 (4.31–8.07)	
Every day	4.53 (3.25–6.31)		2.35 (1.83–3.01)		16.79 (8.50–33.15)		3.83 (2.30–6.38)	
Said something bad about your body shape, size, or appearance								
Never	1.00	<0.001	1.00	<0.001	1.00	<0.001	1.00	<0.001
1–5 times	2.33 (2.14–2.53)		2.18 (2.01–2.38)		2.90 (2.69–3.12)		2.44 (2.26–2.63)	
6+ times	3.93 (3.42–4.50)		3.31 (2.87–3.82)		6.77 (5.99–7.64)		5.06 (4.43–5.77)	
Every day	5.31 (4.27–6.60)		3.35 (2.73–4.11)		14.54 (11.54–18.31)		10.91 (8.06–14.76)	
Asked for personal information over the internet (e.g. address, phone # or last name)								
Never	1.00	<0.001	1.00	<0.001	1.00	<0.001	1.00	<0.001
1–5 times	2.17 (1.95–2.41)		2.16 (1.95–2.39)		3.21 (2.93–3.52)		2.09 (1.91–2.29)	
6+ times	3.31 (2.67–4.12)		2.69 (2.23–3.25)		4.62 (3.82–5.59)		3.66 (3.06–4.37)	
Every day	3.05 (2.07–4.50)		2.56 (1.95–3.36)		6.92 (4.36–10.99)		5.40 (3.22–9.04)	
Made you feel unsafe when you were in contact with them over the internet								
Never	1.00	<0.001	1.00	<0.001	1.00	<0.001	1.00	<0.001
1–5 times	3.50 (2.99–4.09)		3.33 (2.79–3.97)		4.08 (3.62–4.59)		3.12 (2.74–3.55)	
6+ times	6.09 (3.95–9.37)		3.51 (2.29–5.39)		9.66 (6.98–13.37)		6.59 (4.63–9.39)	
Every day	2.89 (1.85–4.50)		1.74 (1.26–2.39)		7.70 (4.35–13.63)		4.62 (2.43–8.80)	
Bullied or picked on you through the internet (e.g. posted something on Facebook or emailed you)								
Never	1.00	<0.001	1.00	<0.001	1.00	<0.001	1.00	<0.001
1–5 times	3.40 (2.99–3.88)		3.22 (2.80–3.69)		3.72 (3.37–4.10)		3.37 (3.01–3.78)	
6+ times	5.53 (4.15–7.37)		3.84 (2.85–5.17)		8.40 (6.90–10.2)		6.44 (5.02–8.25)	
Every day	3.60 (2.48–5.24)		2.20 (1.64–2.95)		19.55 (12.07–31.6)		6.09 (3.71–10.02)	

*Note.* OR = odds ratio; CI = confidence interval; Not reported due to low cell count

## Discussion

While there is a robust literature describing the relationship between general/global assessments of bullying and a variety of detrimental mental health outcomes not only in childhood and adolescence but over the life course (Arseneault 2017; Haraldstad et al. 2016; Moore et al. 2017; Troop-Gordon 2017), there is still much to be understood about diverse and multiple forms of victimization including experiences of discriminatory harassment and contemporary experiences of bullying in an ever-evolving world of cyber technology (Dempsey et al. 2011). The purpose of the current study was to investigate the relationships between specific types of victimization experiences and mental health outcomes among adolescent boys and girls in lower and higher school grades. Findings from this study fully supported our hypothesis that all nine victimization experiences would be associated with higher odds of poorer mental health and feelings of sadness and hopelessness, and that there would be gender- and grade-level differences related to the associations of victimization experiences and mental health outcomes. There was partial support for the dose-response hypothesis among adolescent girls in younger grades but, generally speaking, limited support for a clear, dose-response relationship for boys in younger and older grades.

Taken together, this study advances this field of research with several novel findings. First, all nine different victimization experiences, including the more common forms of bullying like being taunted and ridiculed; discriminatory harassment like having someone speak badly of your sexuality, body composition, or race; and cyber victimization experiences such as feeling unsafe on the internet, being asked to provide personal information, and being bullied online, have a detrimental impact on mental health outcomes. The consistency of these findings across the items is interesting to consider in the context of previous literature, which has suggested that experiences of cyberbullying may operate differently compared to more traditional forms of bullying in terms of intentionality, repetition, and power differentials (Patchin and Hinduja 2015; Volk et al. 2014). It may be the case that these internet-specific victimization experiences may not reflect the common characteristic of the definition of bullying (Freeman et al. 2011; Gladden et al. 2014; Olweus 1996); however, these items are indeed associated with similar poor mental health outcomes, and even relatively low frequencies of all types of victimization experiences (1–5 times or more) were associated with an increased odds of poorer mental health. These findings may prove useful as this field of research continues to evolve, specifically related to how less traditional forms of bullying, including single instances of cyber victimization, are operationalized and measured (Finkelhor et al. 2012). As has been noted by other researchers, a single instance of online bullying can result in a ripple effect of repeated cyber victimization with the potential for devastating mental health consequences (Patchin and Hinduja 2015; Volk et al. 2014; Salmon et al. 2018). These less traditional experiences of peer victimization should be considered in future research.

Second, some interesting gender- and grade-level differences emerged from these data. It is important to acknowledge that previous research has reported mixed findings in terms of bullying experiences, gender, illicit drug use, and depressive symptoms (Cook et al. 2010; Turner et al. 2018). Generally speaking, girls with bullying experiences are more likely to have greater odds of poorer outcomes, including depressive symptoms compared to boys (Williams et al. 2017). Our findings indicate a similar trend that girls had larger odds of poor health outcomes. Although the item used to assess feelings of sadness and hopelessness is not the same as a comprehensive depression measure, previous research has found that girls and women are more likely than boys and men to experience depression (Afifi et al. 2005; Petersen 1991). The current findings indicate that the association between victimization and feelings of sadness and hopelessness may have larger effects among girls, similar to depression – although it should be noted that victimization experiences are still detrimental for boys even if effects are not as large as the effects for girls. One explanation for the gender differences seen in our sample may be due to differences in internalizing and externalizing behaviours symptoms of boys and girls in response to victimization and maltreatment. As noted previously, other studies have suggested gender-specific behavioural pathways in response to maltreatment and victimization, indicating that girls tend to display more internalizing behaviour symptoms, such as sadness and hopelessness, whereas boys tend to exhibit more externalizing behaviours symptoms (Maschi et al. 2008; Nabuzoka et al. 2009). Taken together, these findings provide further evidence that girls and boys experience multiple victimization types, which may have an influence on mental health outcomes.

Along the lines of gender, grade-related differences in experiences of victimization and mental health outcomes are an important avenue of research. This study provides further evidence for the notion that victimization experiences in the lower grades may have a larger association with poor mental health outcomes. Previous research has shown that bullying tends to peak in early adolescence (Hymel and Swearer 2015; Nylund et al. 2007; Salmon et al. 2018; Seo et al. 2017). What this study adds is that the effects of victimization on mental health also seem to be more pronounced among younger (versus older) adolescents. However, the largest grade-level effects found between poor mental health outcomes and victimization experiences were found for girls in lower grades. One potential explanation for this finding may be found in examining children’s coping strategies related to peer victimization experiences. Previous research examined 4th to 8th grade children’s self-reported coping strategies in response to peer victimization and the perceived effectiveness of these strategies. This research found that boys reported using more physical externalizing strategies compared to girls; that girls reported more internalizing, emotion-coping strategies compared to boys; and that, overall, both boys and girls found the various coping strategies to be limited in their efficacy to address peer victimization (Tenenbaum et al. 2011). Future research should endeavour to better understand these important gender- and grade-level differences among victimized adolescents in relation to detrimental outcomes that have the potential to carry on over the life course.

Lastly, perhaps the most novel and interesting findings from this study are related to dose-response trends. While the evidence is clear that all victimization experiences, including feeling unsafe and being asked for personal information over the internet, are associated with poorer mental health outcomes, the dose-response trends based on frequencies of victimization experiences are interesting to interpret. Previous research examining the cumulative and dose-response model among bullying, social supports, and school experiences confirmed that as experiences of victimization increased, positive social supports and school experiences declined (Evans et al. 2014). Our findings were somewhat contradictory to previous research for boys in upper and lower grades. Generally speaking, dose-response trends were more supported for boys in the lower grade for both mental health outcomes, but not for boys in the higher grades. While this is an intriguing finding, it is important not to overlook the fact that *all* specific victimization experiences were significantly associated with poor mental health outcomes for boys at all frequency levels, including the lowest category of ‘1–5 times’. In contrast, dose-response trends were relatively consistent for girls across all grades, indicating that increased frequency of victimization corresponded with greater odds of poorer mental health outcomes. Understanding the increased odds of detrimental outcomes associated with increased frequency of victimization experiences is an important finding that should be used to inform the development and implementation of intervention strategies, which may help reduce the frequency of victimization occurrence for those that may currently be victimized, or those that are at risk of becoming re-victimized.

### Limitations and Implications

The findings from the present study should be considered in the context of a number of important limitations. First, the data were self-reported, which may increase recall and social desirability biases. However, data were collected anonymously, which allows the respondent to respond with more accuracy. Second, the data were cross-sectional in nature and do not allow for inferences regarding causation. Ideally, it may be better to measure victimization at two times or more, and to examine how changes over time are related to mental health outcomes. Third, definitions of bullying in the literature indicate that the experiences are repeated or highly likely to be repeated over time (Freeman et al. 2011; Gladden et al. 2014). In the current study, the response categories were limited (i.e. 'never', '1 to 5 times', '6 or more times', and 'every day' in the past 12 months). Using the higher cut-off level in the current data (i.e. '6 or more times') was thought to be more problematic than the lower cut-off since the higher range may substantially underestimate victimization experiences. Fourth, revisions should be made to the YHS. For example, the mental health assessments in the YHS were limited to mental health functioning and emotional well-being and feelings of sadness and hopelessness. Although the MHC-SF is a valid, reliable, and widely-utilized measure of mental health and well-being, and the indicator of sadness and hopelessness was severe enough to interfere with activities, a broader range of mental health indicators or measures of mental disorders would have improved the study. Additionally, the items used to assess cyber victimization in the YHS including ‘being asked to provide personal information over the internet’ should be expanded and made more specific to better understand adolescents’ experiences of victimization online. Finally, the current study did not include measures of child maltreatment. Previous research indicates that adversity in childhood may be cumulative (Finkelhor et al. 2007), meaning, for example, that children who are victimized in various domains such as school and online may be more likely to also experience child maltreatment at home. Experiencing child maltreatment and other victimization experiences like bullying together may have important implications for understanding the associations with mental health and to better inform both prevention and intervention strategies. In contrast, the home environment may also be protective against bullying victimization (Lereya et al. 2013). Potentially protective home factors were also not available in the data. Examining child maltreatment and peer victimization together is an understudied area of research and should be the focus of future research. Similarly, this study was not able to examine additional individual and family factors such as socioeconomic status, ethnicity, and minority/newcomer status. Future research should consider how these factors may influence the relationship between individual peer victimization experiences and mental health outcomes for younger and older adolescent boys and girls.

Limitations aside, there are some practice and policy implications that may result from consideration of these findings. In terms of supporting children and youth affected by peer victimization, and preventing it from occurring in the first place, it is important to develop and systematically evaluate evidenced-based multi-faceted strategies. Such strategies should not only target more traditional forms of bullying but also address contemporary and anonymized experiences of victimization including cyber victimization and discriminatory harassment given the relationship to poor mental health outcomes. Previous research has suggested that existing school policies and anti-bullying programs have been developed with a focus on traditional forms of bullying without attention to other experiences of peer victimization (Carbone-Lopez, Esbensen & Brick, 2010). A systematic review of anti-bullying interventions concluded that effect sizes were generally small and results were inconsistent and specific to program components including emotional support training, peer counselling and school-based anti-bullying policies (Zych et al. 2015). As such, schools are ideally situated to lead more comprehensive and multi-faceted prevention and intervention efforts but likely need to occur with connections to family and community supports. There is some evidence that a whole-school approach that includes students, parents, classrooms, and entire schools may have favourable outcomes related to effective prevention of bullying, although findings do vary (Menesini and Salmivalli 2017). The findings from the current research support a whole-school approach in the sense that poor mental health outcomes are noted for both boys and girls and across grades 7 to 12. Prevention strategies should include all students and continue across classrooms beginning in the very early grade levels in the context of a developmentally sensitive approach. Importantly, gender and grade differences were noted in the relationships between types of victimization and poor mental health outcomes. These findings may indicate that the prevention strategies should also consider gendered perspectives regarding victimization experiences independently to develop tailored education and support programs. Notably, any newly developed and/or modified peer victimization prevention and intervention strategies should be rigorously evaluated with multi-item assessments that examine differing victimization experiences rather than global, single-item assessments of bullying (Zych et al. 2015). Further work in this area is necessary.

Given these findings suggest that various victimization experiences are associated with poorer mental health outcomes for boys and girls and across younger and older grade categories and that those negative effects remain stable; victims should receive clinical support. Recent clinical guidelines for adolescents involved in bullying suggest that interventions must be multimodal and engage across systems with clients, caregivers, and the school environment; emphasize a client’s subjective experience of victimization through a strengths-based validation approach; prioritize sensitivity and responses to trauma and recognize the traumatic impact victimization and bullying may have; and support the development of the client’s social skills to build self-efficacy, empathy, and effective communication strategies (Byers et al. 2019). More research is needed to determine what methods are effective strategies for preventing and treating victimization for boys and girls separately, and if strategies should evolve according to increasing grade level and child developmental processes.
